# Character

**Published:** 1871-09

**Authors:** 


### CHARACTER.

The word *character*, as commonly employed, means two distinct and oftentimes very dissimilar things. It is sometimes employed to denote the estimation in which we are held by others; and many act according to this notion. They strive to be well esteemed under the idea that, as they succeed in this, they build up character. Now, while a desire to have the good opinion of others is not a low, contemptible motive, and may lead to the peformance of many worthy deeds, it is not the highest. If one has no more elevated spring for his actions than a desire for applause, he cannot preserve his self-respect in any iminent degree. He must be aware, all the while, that he is acting a part—not a base part exactly, if he be willing to act well, to be well esteemed;—but not a part entirely noble. He is very apt—nay, almost certain, if he makes popularity his end—to degrade his dignity, to do that which an enlightened conscience will disapprove. He who follows the fickle fancy of the multitude, may not effect always to act in strict accordance with the laws of rectitude. Reputation then, however good it may be, is not character. It is founded not upon what we are, but upon what we seem, or are supposed, to be. Frequently it is a mere bubble, which a breath has made or may unmake. It is predicated not on the fact that the man has adopted and maintained correct principles, and that he has, in most things, done the best his circumstances allowed, that he has made himself agreeable. One who is solicitous to attain reputation only, need not be careful about being right or consistent. It is his to watch the breezes of popular favor, and trim his sails to suit the changes. It is his concern to ascertain what is expedient, rather than what is right, and he is more anxious to be plausible than to be honest. Character, on the contrary, is what the man is really. It is made up of those principles and qualities which remain when all conventionalities are swept away. The arts of deception which men employ to impose upon others, form no part of the real character, though they sometimes exercise upon it a most unhappy influence. For character is, to a great entent, the result of effort. Nature does indeed, impart to every one a certain cast of intellect and heart. But what this may lead to depends very largely on the individual himself. One may waste the power of his mind until they become worthless or pervert them until they become worse than worthless. So, by disregarding principle and acting always from low groveling motives, one may debase the very best elements of character. With every exercise of virtue the inclination over the evil propensities, renders us more able to sustain another conflict. It is better to have encountered temptation and resisted it than not to have met it at all; for, by the act of successful resistance, the character has been elevated and strengthened. It is by such conquests that a man becomes truly distinguished and glorious.

Thus are we all forever building character. No action or event of our lives fails to exert some influence either for good or evil. When we see one in old age presenting a character resplendent with moral beauty, we must not suppose that he was always thus; nor should we reckon him to have been a reprobate from youth, who seems to have lost all the ennobling capacities of his soul. The former sought to elevate himself. The good that was in him he strove to expand and increase. He endeavored to cultivate his heart by cherishing generous and refining sentiments. As a result of these efforts, his soul become beautiful and the reflection which it threw out to the public gaze awakened love in every beholder. The other, by adopting crooked policies and acting from low motives, habitually depressed the elevating tendencies of his moral nature. Bad passions were allowed to rule in his heart, and soon things wrought a great change, both in his bearing and in his face. The soft lines of gentleness or amiability were effaced, and in their place were the rugged features which wickedness stamps on its devotees. The two pictures, many years ago, were very similar, that they are not so now, is owing to the fact that one has striven to be good and the other has taken equal pains to be wicked.

We have seen it stated by a distinguished author, that childhood is the period when the character is really formed, that youth is the only period of its development. While we believe there is as much in the blood of people as there is in animals, and while we are willing to admit that this difference is the result mostly in the difference of early training, education and association, we do not agree that youth is the only period for the formation and developement of character. If we did, in the present state of things in the Profession, in all parts of the country, we should certainly favor the abolishment of the Ethics, and the turning loose of every individual member to "paddle his own canoe." But believing as we do that the formation and development of character still goes on through youth, and even through much of the period of mature age, perhaps we never reach a state so long as the heart and intellect retain their vitality, when character cannot be materially modified by good or evil influences. With these views, we are hopeful for the Profession—yea, even for many of our prodigal brethren who have wandered long and far from the paths of truth and justice. We have all seen persons who once seem estimable, in whom we can now find nothing to esteem. Again we find those who were once repulsive to us winning more and more of our confidence and affection. We are persuaded that the change is not wholly in us. The character, even of the grown up individual, has grown better or worse. Doubtless the young are more capable of being influenced by circumstances, but the old are seldom wholly unimpressible. Hence, however well we may be acquainted with an individual, we can form very little idea of what he would do in a situation in which he has never been placed. A change of relation will either produce new qualities or develope those which lie latent, and thereby effect a complete modification of the character. If our opinion be correct the consideration of character, the establishment of just laws and the rigid adherance to their requirements are of great importance. There are many persons who seem, under some circumstances, to have all the merits of true character, who, from a change of circumstances, will assume a position which will prove them to be perfectly destiute of character, and possessed of a constituted selfishness, which no training or teaching can change, and nothing but the strong arm of the law and a just indignation of public opinion can influence.

A good name is very desirable—more to be sought than wealth, or power, or honor. But if good reputation be valuable, how infinitely more valuable is a good character—the possession of the substance of which the former is the mere shadow. If it gives us pleasure to be praised by others, how much more pleasure shall it give when we are able to approve ourselves—to feel in our hearts that we are true and worthy. Reputation is indeed worth effort; for it will give us an inward delight, and increase our usefulness. But far more than reputation, and even at the risk of loosing reputation, should we seek to have a mind conscious of its own rectitude, and a character well established on the immutable principles of truth and justice.

